# *BEST1* sequence variants in Italian patients with vitelliform macular dystrophy

**Published:** 2012-11-17

**Authors:** Andrea Sodi, Ilaria Passerini, Vittoria Murro, Roberto Caputo, Giacomo Maria Bacci, Mirela Bodoj, Francesca Torricelli, Ugo Menchini

**Affiliations:** 1 Department of Specialized Surgical Sciences, Eye Clinic, University of Florence, Italy; 2 Department of Genetic Diagnosis, Azienda Ospedaliero-Universitaria Careggi, Florence, Italy; 3 Pediatric Ophthalmology Unit, Azienda Ospedaliero-Universitaria Meyer, Florence, Italy

## Abstract

**Purpose:**

To analyze the spectrum of sequence variants in the *BEST1* gene in a group of Italian patients affected by Best vitelliform macular dystrophy (VMD).

**Methods:**

Thirty Italian patients with a diagnosis of VMD and 20 clinically healthy relatives were recruited. They belonged to 19 Italian families predominantly originating from central Italy. They received a standard ophthalmologic examination, OCT scan, and electrophysiological tests (ERG and EOG). Fluorescein and ICG angiographies and fundus autofluorescence imaging were performed in selected cases. DNA samples were analyzed for sequence variants of the *BEST1* gene by direct sequencing techniques.

**Results:**

Nine missense variants and one deletion were found in the affected patients; each patient carried one mutation. Five variants [c.73C>T (p.Arg25Trp), c.652C>T (p.Arg218Cys), c.652C>G (p.Arg218Gly), c.728C>T (p.Ala243Val), c.893T>C (p.Phe298Ser)] have already been described in literature while another five variants [c.217A>C (p.Ile73Leu), c.239T>G (p.Phe80Cys), c.883_885del (p.Ile295del), c.907G>A (p.Asp303Asn), c.911A>G (p.Asp304Gly)] had not previously been reported. Affected patients, sometimes even from the same family, occasionally showed variable phenotypes. One heterozygous variant was also found in five clinically healthy relatives with normal fundus, visual acuity and ERG but with abnormal EOG.

**Conclusions:**

Ten variants in the *BEST1* gene were detected in a group of individuals with clinically apparent VMD, and in some clinically normal individuals with an abnormal EOG. The high prevalence of novel variants and the frequent report of a specific variant (p.Arg25Trp) that has rarely been described in other ethnic groups suggests a distribution of *BEST1* variants peculiar to Italian VMD patients.

## Introduction

Best vitelliform macular dystrophy (OMIM # 153700; VMD) is a macular disease, which generally appears in childhood with a yellowish yolk-like lesion in the macula [1-3]. The first pedigree was described by Best in 1905 [4].

The disease is associated with the accumulation of lipofuscin at the level of the retinal pigment epithelium (RPE); over time the central yellow lesions progressively disintegrate and macular atrophy or fibrosis often develops [1,2]. The clinical picture evolves over many years producing a gradual decline of visual acuity; according to Gass [1] the macular lesions progress through various well defined stages: vitelliform, pseudohypopyon, vitelliruptive (scrambled egg), atrophic and cicatricial. Some patients develop choroidal neovascularization [1], which can be treated with photodynamic therapy [5,6] or intravitreal antiangiogenic drugs [7,8]. In the large majority of families the electro-oculogram (EOG) is markedly abnormal in all stages of progression and in phenotypically normal carriers [9], even if several studies report some patients with normal EOG [10-12]. Full-field ERG is usually normal [10,11] while mfERG often presents reduced amplitudes [13]. Fundus autofluorescence (FAF) imaging shows an increased autofluorescence of the vitelliform deposits [14] while OCT allows the visualization of the vitelliform substance and the associated alterations of the RPE and of the photoreceptors [15-17].

VMD is inherited as an autosomal dominant trait, with incomplete penetrance and highly variable clinical expression. It has been associated with alterations in the gene *BEST1* (OMIM # 607854; previously known as *VMD2*), mapped to the long arm (q13) of chromosome 11, encoding the 585-amino acid transmembrane protein bestrophin-1 [18,19]. This protein localizes to the basolateral membrane of RPE cells [20] and functions as a chloride channel [21,22] but it may act as an inhibitor of intracellular voltage-dependent Ca^2+^ channels, too. It has also been involved in pH and cell volume regulation and a possible role as a HCO_3_^-^ channel has been proposed too [23]. Bestrophin-1 dysfunction results in abnormal fluid and ion transport by the RPE, determining a weakened interface between RPE and photoreceptors; this may affect retinoid transport and photoreceptors outer segment phagocytosis by the RPE, finally leading to increased lipofuscin deposition [24].

In addition to VMD, *BEST1* variants have been associated with several other eye diseases [22] including adult-onset vitelliform macular dystrophy [25], autosomal recessive bestrophinopathy [26-28], autosomal dominant vitreoretinochoroidopathy [29,30], retinitis pigmentosa [31], microcornea, retinal dystrophy, cataract, and posterior staphyloma (MRCS syndrome) [32]. To date more than 150 *BEST1* variants have been identified in VMD [22] (mutation-database; Retina international, databases accessed January 2012); the large majority of them are missense variants [22,33,34] resulting in amino acid changes in the N-terminal part of the protein.

*BEST1* variants have been investigated in several different ethnic groups and in isolated Italian families [6,12,35] but at present there is no specific study on *BEST1* variants in Italian VMD patients. Recently the results were published [36] of a *BEST1* molecular analysis performed on a group of 23 patients, 10 of whom were from three Italian families. In the present study sequence variants of the *BEST1* gene were determined in a group of Italian patients affected by VMD; these 30 patients were taken from a large sample of 19 Italian families, predominantly originating from central Italy.

## Methods

### Clinical evaluation

Nineteen Italian families, of which at least one family member was affected by VMD, were recruited through the Hereditary Retinal Degenerations Referring Center of the Eye Clinic of the University of Florence. Criteria for the Best phenotype included the following: 1) juvenile-to-adult onset of the disease; 2) bilateral macular dystrophy with the typical round lipofuscin lesions at the posterior pole (including patients with different stages of the disease); 3) normal ERG; 4) abnormal EOG with Arden ratio always below 1.50. We also studied some apparently healthy relatives of the patients who agreed to participate to identify possible asymptomatic carriers. The study adhered to the tenets of the Declaration of Helsinki and was approved by the Local Ethics Committee. Moreover, each patient gave written informed consent.

All the subjects included in the study were clinically evaluated by means of a standard ophthalmologic examination, fundus photography, OCT scan (Topcon 3D OCT-1000, Topcon Medical Systems Inc., Oakland, NJ) and electrophysiological tests (EOG, ERG; Electrophysiological Diagnostic Unit Retimax, Roland Consult, Brandenburg, Germany) performed according to the existing ISCEV Guidelines [37,38]. In most of the cases electrophysiological examinations were performed in our Department but in two patients we accepted examinations performed during the previous year in other hospitals and included in the medical documentation of the patient. Fluorescein angiography (FA; Zeiss Retinograph with Image Processing Software Visupac, Carl Zeiss, Dublin, CA) was performed on eight patients: to improve the diagnosis (one case), to investigate the possible presence of choroidal neovascularization (CNV) in patients complaining of reduced visual acuity and/or metamorphopsia (six cases), and on one patient presenting retinal vein occlusion. We also took into consideration three fluorescein angiographies performed in other Hospitals. ICG imaging was performed on six patients for a more refined diagnosis of possible CNV. Fundus Autofluorescence Imaging (FAF; Confocal SLO, HRA Inc., Heidelberg Engineering, Heidelberg, Germany) was performed on all the affected patients who agreed to collaborate (18 patients).

### DNA extraction and PCR amplification

Following informed consent and a complete medical history of each family, 10 ml of peripheral blood were obtained from the antecubital vein using EDTA-containing vials. DNA was extracted from 200 μl of peripheral blood using an automated method involving the BioRobot EZ1 workstation (QIAGEN GmbH, Germany).

The PCR amplification of 11 exons and flanking intronic regions of the *BEST1* gene was performed using the Core System-Robotic Station (Beckman Coulter Inc., Miami, FL). PCR products were purified by the Biomek NX station (Beckman Coulter).

### Mutational analysis

Standard cycle-sequencing reaction with BigDye terminator mix v1.1 (Applied Biosystems, Foster City, CA) contained 3–10 ng purified PCR products in 20 μl and were performed with forward and reverse primers used for initial amplification. The sequencing reactions were precipitated, dried and then sequenced on a sequencer 3730 DNA Analyzer. Finally, data obtained from the Sequence Analysis Software (Applied Biosystems) were aligned with the wild-type *BEST1* gene sequence (GenBank Database;). DNA samples of the probands were analyzed for mutations in all the 11 exons of the *BEST1* gene by direct sequencing [28]; in the other members of the family, either clinically healthy or affected, *BEST1* gene sequencing was limited to the exon in which the mutation was detected in the proband. A sequence mismatch was considered a potential disease-causing variant only if absent in 300 healthy controls, associated with amino acidic change, and confirmed by a new independent PCR (EMQN Best Practice Guidelines)

The Alamut-1.5 software (Interactive Biosoftware, Rouen, France) was used to predict the impact of unclassified variants on the protein function; Alamut is a software suite dedicated to sequence variants interpretation and the prediction of pathogenicity, assembling the information provided by three scoring systems (PolyPhen, SIFT, Align GVGD) on the basis of several parameters such as biophysical characteristics of amino acids and their conservation across species (EuroGenTest) [39-41].

## Results

Thirty Italian patients with a diagnosis of VMD (from 19 independent pedigrees) were clinically examined: 19 were male and 11 female. The mean age was 42.5 years (±19.1 years; range 11–82 years). The Snellen visual acuity ranged from 0.1 to 1.0, with an average value of 0.62 (±0.31). In 18/30 patients (60%) best corrected visual acuity (BCVA) was different (2 lines or greater) between the two eyes. In the eyes with the better visual acuity, BCVA was equal to or better than 0.5 in 12/13 (92.3%) of the patients younger than 40 years of age, and in 13/17 (76.4%) of the patients older than 40 years of age. In the eyes with the worse visual acuity, BCVA was equal to or better than 0.5 in 7/13 (53.8%) of the patients younger than 40 years of age and in 6/17 (35.2%) of the patients older than 40 years of age.

**Figure 1:**
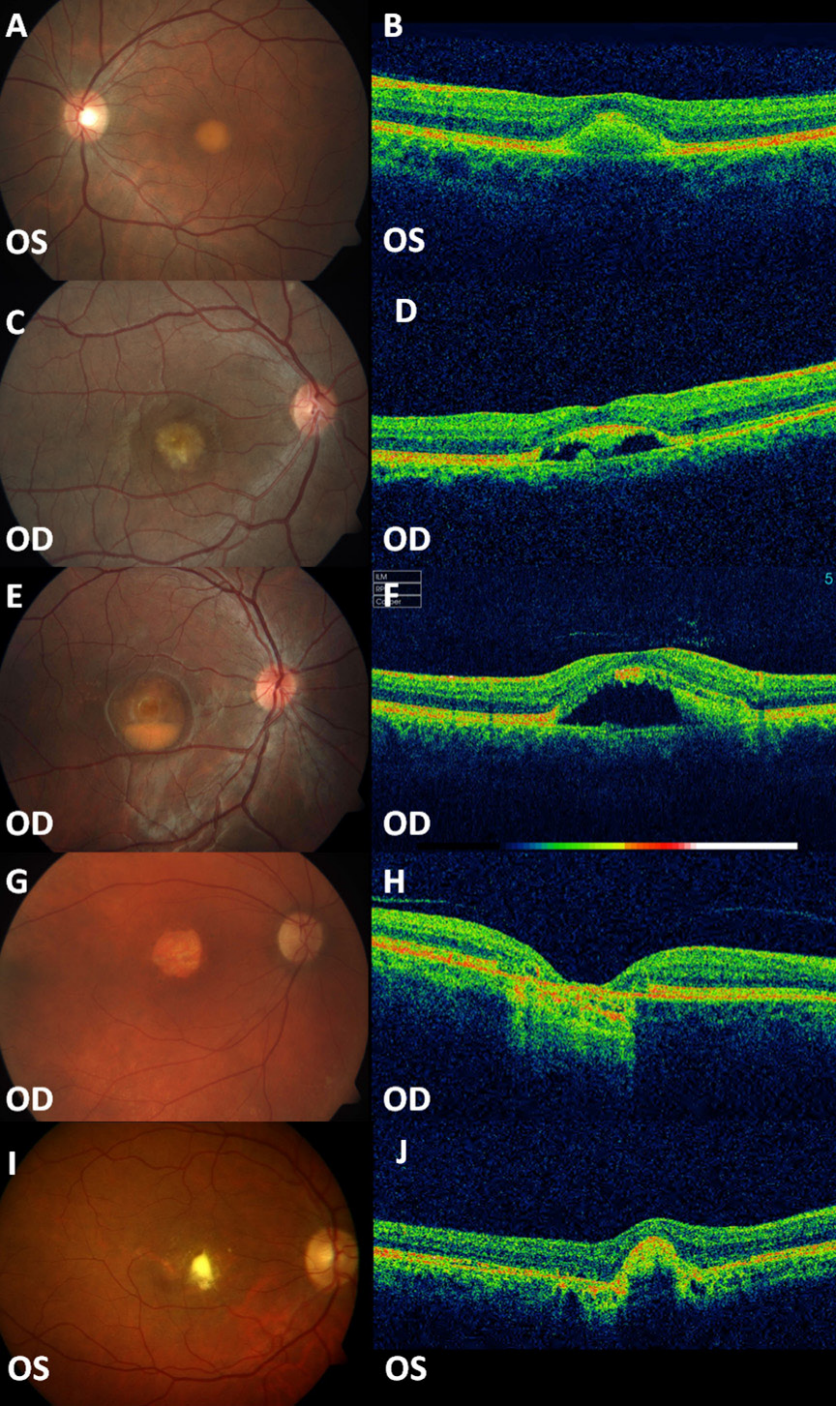
Different stages of VMD (Fundus photographs and OCT). **A**, **B**: Vitellifom disc (Patient R-II-1). **C**, **D**: Vitelliruptive stage (Patient C-II-1). **E**, **F**: Pseudohypopyon stage (Patient B-II-1). **G**, **H**: Macular atrophy (Patient **Q**-**I**:-1). **I**, **J**: Macular fibrosis (Patient I-II-1). OD represents the right eye, OS represents the left eye.

All the patients showed fundus lesions according to the typical stages as described by Gass (Figure 1) [1]: 11 eyes could be classified as vitelliform lesions, 8 eyes as pseudohypopion stage, 16 as vitelliruptive stage, 13 as macular atrophy, and 11 as fibrotic macular scar. Stage classification was the same for both eyes in 18 patients and asymmetric in 12 patients. One patient showed a vitelliform lesion in one eye while the macula of the fellow eye presented only mild RPE dystrophy. Clinical data of our series are summarized in Table 1 and Table 2.

**Table 1:** Clinical details of the VMD patients included in the study (families A-G).

Family	Family member	Sex	Age (years)	Onset (years)	BCVA OD	BCVA OS	Fundus OD	Fundus OS	Notes
A	III-3	F	11	6	0.8	1.0	VL	PV	Unil
A	II-5	M	45	//	1.0	1.0	Norm	Norm	
A	II-4	F	42	//	1.0	1.0	Norm	Norm	
A	III-4	M	9	//	1.0	1.0	Norm	Norm	
A	II-3	F	42	//	0.6	1.0	Norm	Norm	Ambl OD
A	III-1	M	14	//	1.0	1.0	Norm	Norm	
A	III-2	F	12	//	1.0	1.0	Norm	Norm	
A	I-2	F	78	//	0.5	1.0	GC	GC	Glau OU
B	II-1	M	16	10	0.7	0.9	PH	PH	
B	I-1	M	44	39	0.9	1.0	VR	VR	
B	I-2	F	40	//	1.0	1.0	Norm	Norm	
C	II-1	M	18	8	0.8	0.8	VR	VR	CNV; P
C	I-1	M	54	//	1.0	1.0	Norm	Norm	
C	I-2	F	50	//	1.0	1.0	Norm	Norm	
D	II-1	M	22	14	0.3	1.0	FI	VL	CNV
D	I-1	M	55	//	1.0	1.0	Norm	Norm	
D	I-2	F	57	35	0.4	0.3	AT	AT	
E	I-1	M	62	25	0.2	0.2	AT	AT	
E	II-1	M	42	//	1.0	1.0	Norm	Norm	
F	II-1	F	46	10	0.2	0.2	AT	AT	
G	I-2	M	60	29	0.3	0.1	FI	FI	
G	II-1	M	27	8	1.0	1.0	VL	VL	
G	II-2	F	25	7	1.0	1.0	VL	VL	
G	I-3	M	68	35	0.8	0.9	AT	VL	Mult OS
G	II-5	F	28	//	1.0	1.0	Norm	Norm	

For each patient the stage of disease is indicated. (BCVA: Best Corrected Visual Acuity; VL: vitelliform lesion; PV: pre-vitelliform lesion; GC: glaucomatous cupping; PH: pseudohypopyon stage; VR: vitelliruptive stage; AT: atrophic stage; FI: fibrotic stage; CNV: previous choroidal neovascularization; P: treated with photodynamic therapy; Unil: unilateral presentation of the disease; Mult: multifocal presentation of the disease; Ambl: Ambliopia; Glau: Glaucoma; OD: right eye; OS: left eye; //: unknown; OU: both eyes).

**Table 2:** Clinical details of the VMD patients included in the study (families H-S).

Family	Family Member	Sex	Age (years)	Onset (years)	BCVA OD	BCVA OS	Fundus OD	Fundus OS	Notes
H	I-1	M	63	53	0.4	0.8	VR	PH	MultRE
H	II-1	M	34	//	1.0	1.0	Norm	Norm	
H	II-2	F	27	//	1.0	1.0	Norm	Norm	
I	II-1	M	22	2	0.3	0.7	FI	FI	
I	I-1	M	60	30	0.8	1.0	VL	VL	MultOU
I	II-2	F	20	//	1.0	1.0	Norm	Norm	
J	III-1	F	15	5	0.4	0.8	FI	FI	CNV; P
J	II-2	M	46	6	0.5	0.5	VR	VR	
J	II-3	M	52	10	0.7	0.3	FI	FI	
J	I-1	M	82	50	0.8	0.1	AT	AT	
K	I-2	F	45	33	0.3	0.7	FI	VR	CNV; P; A
K	II-1	M	16	7	0.2	0.9	FI	PH	CNV;P
K	I-3	F	40	//	1.0	1.0	Norm	Norm	
K	II-2	M	21	//	0.6	0.6	Norm	Norm	Cong Ny
K	II-3	F	19	//	1.0	1.0	Norm	Norm	
L	II-1	M	45	15	0.4	0.6	PH	VR	
M	II-1	F	38	25	0.3	0.5	VR	VR	
N	III-1	M	35	25	1.0	0.9	PH	VR	
N	II-3	M	47	35	0.2	1.0	AT	PH	
O	II-1	F	48	35	0.1	0.1	VR	VR	
P	II-1	F	62	47	0.5	0.6	VR	PH	
Q	I-2	F	72	60	0.2	0.5	AT	VL	
Q	II-1	F	44	//	1.0	1.0	Norm	Norm	
R	II-1	F	41	37	0.6	0.9	VR	VL	
S	II-1	M	32	25	0.8	1.0	AT	AT	

For each patient the stage of disease is indicated. (BCVA: Best Corrected Visual Acuity; VL: vitelliform lesion; PH: pseudohypopyon stage; VR: vitelliruptive stage; AT: atrophic stage; FI: fibrotic stage; CNV: previous choroidal neovascularization; P: treated with photodynamic therapy; A: treated with intravitreal bevacizumab; Mult: multifocal presentation of the disease; Cong Ny: congenital nystagmus; OD: right eye; OS: left eye; //: unknown; OU: both eyes).

EOG was always abnormal with Arden ratio lower than 1.50 while ERG was always within normal limits. Electrophysiological results are summarized in Table 3 and Table 4.

**Table 3:** Electrophysiological and molecular results of the VMD patients included in the study (families A-G). For each eye Arden ratio is indicated.

Family	Family member	EOG OD	EOG OS	Allele1	Allele2
A	III-3	1.22	1.40	p.Arg218Gly	WT
A	II-5	2.00	2.10	WT	WT
A	II-4	1.40	1.45	p.Arg218Gly	WT
A	III-4	NA	NA	p.Arg218Gly	WT
A	II-3	1.47	1.42	p.Arg218Gly	WT
A	III-1	NA	NA	p.Arg218Gly	WT
A	III-2	2.15	1.95	WT	WT
A	I-2	NA	NA	p.Arg218Gly	WT
B	II-1	1.30	1.25	p.Arg218Cys	WT
B	I-1	1.40	1.20	p.Arg218Cys	WT
B	I-2	2.13	2.35	WT	WT
C	II-1	1.35	1.25	p.Arg25Trp	WT
C	I-1	1.32	1.11	p.Arg25Trp	WT
C	I-2	2.45	2.30	WT	WT
D	II-1	1.40	1.40	p.Phe298Ser	WT
D	I-1	2.11	2.45	WT	WT
D	I-2	1.35	1.25	p.Phe298Ser)	WT
E	I-1	1.43	1.30	p.Arg25Trp	WT
E	II-1	NA	NA	p.Arg25Trp	WT
F	II-1	1.15	1.20	PIle73Leu	WT
G	I-2	1.30	1.45	p.Arg25Trp	WT
G	II-1	1.28	1.33	p.Arg25Trp	WT
G	II-2	1.45	1.32	p.Arg25Trp	WT
G	I-3	1.34	1.15	p.Arg25Trp	WT
G	II-5	2.30	2.45	WT	WT

**Table 4:** Electrophysiological and molecular results of the VMD patients included in the study (families H-S). For each eye Arden ratio is indicated.

Family	Family Member	EOG OD	EOG OS	Allele1	Allele2
H	I-1	1.40	1.42	p.Arg25Trp	WT
H	II-1	1.55	1.48	p.Arg25Trp	WT
H	II-2	1.50	1.55	p.Arg25Trp	WT
I	II-1	1.25	1.30	p.Arg25Trp	WT
I	I-1	1.45	1.40	p.Arg25Trp	WT
I	II-2	1.93	2.10	WT	WT
J	III-1	1.15	1.22	p.Arg218Cys	WT
J	II-2	1.48	1.35	p.Arg218Cys	WT
J	II-3	1.23	1.45	p.Arg218Cys	WT
J	I-1	1.25	1.15	p.Arg218Cys	WT
K	I-2	1.14	1.02	p.Arg218Cys	WT
K	II-1	1.20	1.15	p.Arg218Cys	WT
K	I-3	2.10	2.39	WT	WT
K	II-2	2.22	2.10	WT	WT
K	II-3	2.25	2.40	WT	WT
L	II-1	1.30	1.09	p.Arg218Cys	WT
M	II-1	1.23	1.41	p.Ile295del	WT
N	III-1	1.40	1.45	p.Asp304Gly	WT
N	II-3	1.49	1.32	p.Asp304Gly	WT
O	II-1	1.18	1.22	p.Ala234Val	WT
P	II-1	1.36	1.26	p.Arg25Trp	WT
Q	I-2	1.23	1.43	p.Arg25Trp	WT
Q	II-1	2.51	2.80	WT	WT
R	II-1	1.45	1.48	p.Phe80Cys	WT
S	II-1	1.34	1.42	p.Asp303Asn	WT

OCT showed the accumulation of hyperreflective material in different stages of disorganization between the neuroretina and the RPE, and allowed the visualization of fibrotic or atrophic macular alterations. None of the patients showed any sign of new vessels on fundus examination and OCT scans, or on FA and ICG when performed. In previous years five patients had been diagnosed with CNV; four patients showed sudden visual loss and macular hemorrhage while another patient presented a more intriguing clinical picture with sudden visual loss, very mild ophthalmoscopic changes and subtle FA and ICG leakage. One patient (who developed CNV in 2001) was not treated, three patients received photodynamic therapy (PDT), and one patient was treated with PDT and intravitreal bevacizumab; the three patients treated with PDT showed first a stabilization of the clinical picture and then a slow improvement of visual acuity.

All the 20 asymptomatic relatives showed a normal fundus appearance, normal visual acuity, and ERG response. Ten subjects showed a normal EOG response while five subjects showed an abnormal EOG with reduced Arden ratio. Five subjects were not available for electrophysiological testing.

Ten different *BEST1* sequence variants were identified in the 30 VMD patients; each family was found to have a specific *BEST1* variant that segregated with the disease. Five of these variants [c.73C>T (p.Arg25Trp), c.652C>T (p.Arg218Cys), c.652C>G (p.Arg218Gly), c.728C>T (p.Ala243Val), c.893T>C (p.Phe298Ser)] have already been described in literature (mutation-database; Retina international, databases accessed January 2012), while five variants [c.217A>C (p.Ile73Leu), c.239T>G (p.Phe80Cys), c.883_885del (p.Ile295del), c.907G>A (p.Asp303Asn), c.911A>G (p.Asp304Gly)] have not previously been reported, and were not detected in 150 unaffected control individuals (300 chromosomes) of Italian origin. The variant p.Ile295del has already been reported [11,18] but the variant detected in our series shows a slight difference to the sequence change already described in literature; in fact in our patient the deleted codon is ATC while in the previous reports it is TCA.

Nine variants were missense, and only one was a deletion. All the patients carried a single variant on one allele. One heterozygous variant was also identified in all of the five clinically healthy relatives with normal fundus, visual acuity and ERG, but with abnormal EOG; they were members of three different families and were heterozygous for one of two variants already previously described: p.Arg218Gly was detected in two members of the same family, while p.Arg25Trp was found in three subjects from two different families. Another four clinically unaffected subjects carried one *BEST1* variant but were not available for EOG examination.

**Figure 2:**
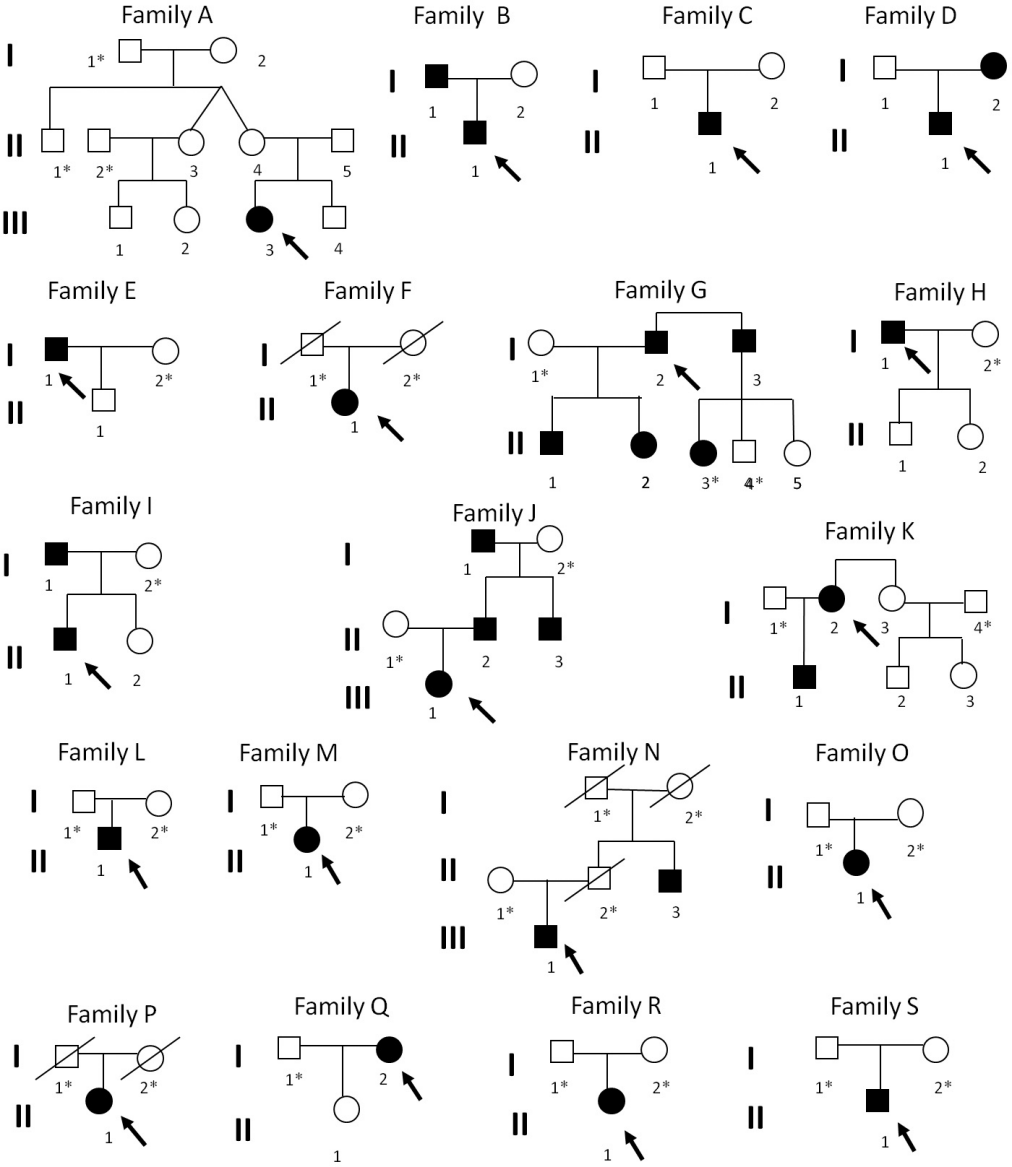
Pedigrees of the families included in the study, showing the autosomal dominant mode of inheritance of VMD. The asterisk * indicates the family members who have not been clinically examined; in these cases the classification affected/not affected is based on the proband’s report.

All the other family members who showed a completely normal phenotype presented a wild-type genotype on both *BEST1* alleles. The pedigrees of the families included in the study are shown in Figure 2. The *BEST1* sequence variants detected in all the subjects who were available for genetic testing are summarized in Table 3 and Table 4.

p.Arg218Cys and p.Arg25Trp were the most common allelic variants among our VMD patients of Italian origin. p.Arg218Cys was found in 5/19 families (26.3%) and in 12/30 (40%) patients; p.Arg25Trp was found in 7/19 families (36.8%) and in 10/30 patients (33.3%). Table 5 shows the theoretical consequences calculated by the Alamut software (Interactive Biosoftware, database accessed January 2012) of four of the five novel variants; note that its scoring systems provide a predictive evaluation only for missense variants.

**Table 5:** Novel *BEST1* variant characterization by means of the Alamut software.

cNomen	Exon	pNomen	Amino acid conservation	AGVGD class	SIFT (score)	POLYPHEN (score)
c.217A>C	3	p.Ile73Leu	Moderately	C0	Tolerated (0.93)	Benign (1.274)
c.239T>G	3	p.Phe80Cys	Highly	C65	Affected protein function (0.00)	Benign (2.771)
c.907G>A	8	p.Asp303Asn	Highly	C15	Affected protein function (0.00)	Possibly damaging (1.960)
c.911A>G	8	p.Asp304Gly	Highly	C65	Affected protein function (0.00)	Probably damaging (2.530)

Predictions of the scoring methods (Align GVGD, SIFT, Polyphen) are shown.

## Discussion

We screened a group of 30 Italian VMD patients and some of their relatives for *BEST1* sequence variants. In the affected patients BCVA was variable (ranging from 0.1 to 1.0) showing a significant difference between the two eyes in the majority of cases, and was more severely reduced in older subjects. These results are consistent with those of a previous study investigating visual impairment in VMD [42].

We reported one *BEST1* variant in all the VMD patients. This high detection rate is in agreement with other studies performed on different ethnic groups [22,36,43-45] and confirms the strong association between VMD phenotype and *BEST1* sequence variants.

Most of the variants (9/10) were missense and only one was a deletion (p.Ile295del). The high incidence of *BEST1* missense variants in VMD is consistent with the mutation spectrum of the gene databases (mutation-database; Retina international). These findings have already been reported in previous studies [22,33,34,44,46] raising the hypothesis of a possible dominant negative effect of the abnormal protein to determine the VMD phenotype. As in other ethnic groups, the majority (8/10) of the identified variants are clustered in four regions frequently affected by variants and probably associated with a relevant functional role [33,34]. Four out of ten variants were located in the protein region between 289 and 310 aa, recently reported to harbor a significant number of sequence variants [47].

In our series of Italian patients, mainly originating from central Italy, five of the 10 reported variants have never been previously described. All the five variants occurred in moderately to highly conserved regions of the protein. Four are missense and one (Ile295del) is a deletion.

Three of the missense variants (p.Phe80Cys, p.Asp303Asn, p.Asp304Gly) lead to an amino acid group change with different physicochemical properties and are labeled as affecting protein function by the SIFT algorithm of the Alamut software. p.Asp303Asn and p.Asp304Gly lie very close to one another in a region where other variants have already been reported [18,35], suggesting a significant pathogenic impact for the alterations located in this region of the protein.

The physiopathological effect is more questionable for the other missense variant (p.Ile73Leu) because it is associated with a change between amino acids that are chemically similar, and it is interpreted as a benign or tolerated substitution by the software; moreover, p.Ile73Leu is located outside the four regions frequently affected by mutations. However, its association with a typical VMD phenotype (Patient F-II-1) suggests a pathogenic role.

**Figure 3:**
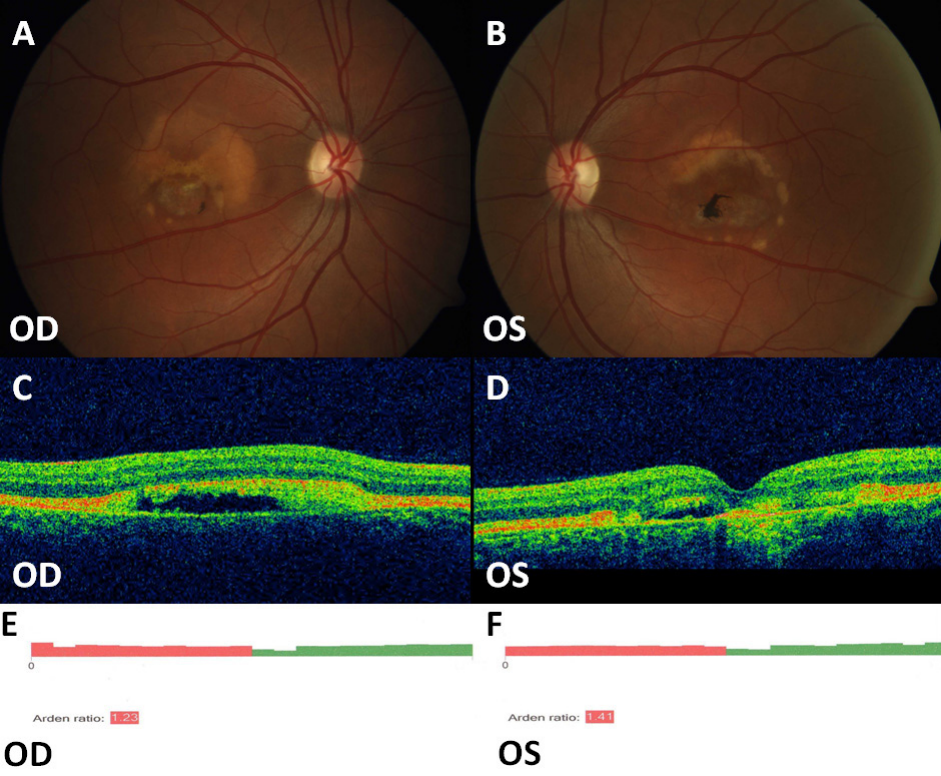
Fundus photographs, OCT and EOG of Patient M-II-1 carrying the novel sequence variant p.Ile295del. **A**, **B**: Vitelliform lesion with partial re-absorption of the vitelliform material. **C**, **D**: Macular detachment of the neurosensory retina partially occupied by hyperreflective material. **E**, **F**: Reduced Arden Test. OD represents the right eye, OS represents the left eye.

The p.Ile295del deletion found in our series is the deletion of codon ATC, different from codon TCA whose deletion has already been reported [11,18]. However both nucleotide variants lead to the same variation of the protein, with the elimination of an isoleucine and an alteration of the protein sequence that is likely to impair its function. This variant does not lead to a premature stop codon in the downstream sequence of the protein, in agreement with the in vitro study showing a dominant negative effect of p.Ile295del on Cl^–^ channel function [48]. In a previous paper [11] the variant p.Ile295del with the deletion of codon TCA was associated with reduced penetrance and normal EOG in the early stages of the disease; in our series the patient (patient M-II-1) carrying the variant p.Ile295del with deletion of codon ATC showed a relatively severe phenotype with reduced visual acuity, abnormal EOG, and bilateral macular lesions in a vitelliruptive stage (Figure 3).

Two variants (p.Arg218Cys and p.Arg25Trp) are highly prevalent among our VMD patients and clinically normal individuals with an abnormal EOG. In our study p.Arg218Cys has been identified in 4/19 families (21%) and in 9/30 patients (30%); Arginine in 218 position is a well known mutational hotspot, suggesting that this site may also have a particular structural relevance for bestrophin function [10,33,43,44,46,49,50]. p.Arg25Trp has been found in 6/19 families (31%) and in 11/30 patients (36%); it has already been reported [6,34,36,47,51], but its high frequency in our group suggests that it may be a recurrent variant in the Italian population.

Recently 10 Italian VMD patients from 3 families were screened for *BEST1* mutations [46]; among the three detected variants two were not found in our series while the third (p.Ala243Val) was identified in one of our patients, too. The variant p.Ala243Val has been reported in association with a mild VMD phenotype and a normal or near-normal EOG [25,36,52]; on the contrary, our patient carrying the same variant (patient O-II-1) showed a severe clinical picture with BCVA reduced to 0.1 in both eyes, abnormal EOG and bilateral neuroretinal detachment at the posterior pole, even though her symptoms appeared relatively late (35 years of age).

**Figure 4:**
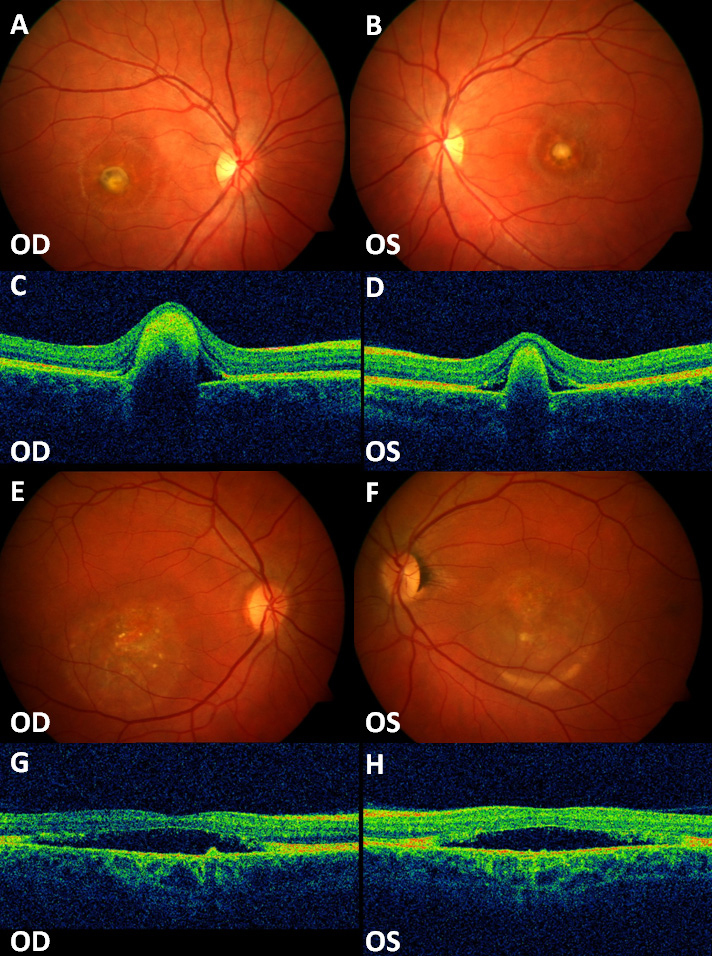
Fundus photographs and OCT of patients J-III-1 (proband) and J-II-2 (proband’s father) showing intrafamilial phenotypic variability. **A**, **B**, **C**, **D**: Fibrotic lesion at the posterior pole centered in the macula; the patient received bilateral PDT treatment for CNV (Patient J-III-1). **E**, **F**, **G**, **H**: Vitelliruptive stage (Patient J-II-2). OD represents the right eye, OS represents the left eye.

Affected members, sometimes even within the same family, occasionally showed variable phenotypes (different ages of onset, different visual loss severity, and fundus appearance). Figure 4 shows the different phenotypes of two members of the same family.

Five patients, carrying three different allelic variants: p.Arg25Trp, p.Arg218Cys, p.Phe298Ser, developed CNV during the course of the disease.

Three patients from three independent pedigrees, all carrying the same variant, p.Arg25Trp, showed multifocal vitelliform lesions in at least one eye; however, p.Arg25Trp is one of the most common variants in our series, and other affected family members do not show a multifocal clinical picture so we cannot speculate a genotype-phenotype correlation.

Variable expressivity of *BEST1* mutations has already been reported [22,36], and may be due to the influence of environmental factors or unknown modifier genes [22]; this suggests that in families affected by VMD molecular genetic results must be interpreted with caution especially when providing genetic counseling. We found one of the two variants p.Arg25Trp and p.Arg218Gly in all of the five asymptomatic carriers with EOG abnormalities without overt clinical alterations; however, our series is too small to establish a different penetrance of the various genotypes.

In conclusion, we identified five novel and five previously reported *BEST1* sequence variants in a series of 30 VMD patients from 19 independent Italian families; to our knowledge this is the largest study in the literature investigating *BEST1* variants in an Italian population. The high number of novel variants and the high prevalence in our patients of a variant that is uncommon in other groups of different origin (p.Arg25Trp) suggest a difference in the spectrum of *BEST1* sequence variants between Italian VMD patients and other ethnic groups. A better knowledge of the spectrum of *BEST1* sequence variants in specific populations may help to improve molecular diagnostic approaches and select patients for future therapeutic options.
